# The moderating role of techno-related control in the relationship between algorithmic management and technostress among sport science professionals

**DOI:** 10.3389/fpubh.2026.1972493

**Published:** 2026-09-25

**Authors:** Tayfun Kara, Oğuzhan Gül, Yunus Şahinler, Furkan Çamiçi, Orhan Gök, Muzaffer Toprak Keskin, Mahmut Ulukan, Mehmet Bayansalduz, Faik Öz

**Affiliations:** 1 Department of Sport Management, Faculty of Sport Sciences, Kahramanmaraş Sütçü İmam University, Kahramanmaraş, Türkiye; 2 Department of Health, Culture and Sports, Sivas Cumhuriyet University, Sivas, Türkiye; 3 Department of Exercise and Sport Sciences, Faculty of Sport Sciences, İstanbul Gelişim University, İstanbul, Türkiye; 4 Division of Movement and Training Sciences, Department of Coaching Education, Faculty of Sport Sciences, Hitit University, Çorum, Türkiye; 5 Department of Sport Management, School of Physical Education and Sports, Osmaniye Korkut Ata University, Osmaniye, Türkiye; 6 Department of Coaching Education, Faculty of Sport Sciences, Nevşehir Hacı Bektaş Veli University, Nevşehir, Türkiye; 7 Department of Recreation, Faculty of Sports Sciences, Istanbul Gelisim University, İstanbul, Türkiye; 8 Department of Sport Management, Faculty of Sport Sciences, İstanbul Topkapı University, İstanbul, Türkiye; 9 Department of Exercise and Sport Sciences, Faculty of Sport Sciences, Avrasya University, Trabzon, Türkiye

**Keywords:** algorithmic management, moderation, sport science professionals, techno-related control, technostress

## Abstract

**Introduction:**

The increasing integration of digital and artificial intelligence-supported systems into sport organizations has transformed managerial processes such as performance monitoring, evaluation, planning, scheduling, and decision-making. This study examined the association between algorithmic management and technostress among sport science professionals and investigated the moderating role of techno-related control.

**Methods:**

A quantitative cross-sectional design was employed with 372 sport science professionals working in sport-related organizations where digital or AI-supported systems were used to perform or support managerial functions. Algorithmic management was assessed using the Algorithmic Management Questionnaire, technostress was measured using the Turkish version of the Technostress Creators Scale, and techno-related control was assessed using the Techno-related Control subscale of the Technology-Related Moderator Scale. The measurement model was evaluated through confirmatory factor analysis, and the hypothesized moderation model was tested using Hayes’ PROCESS Macro Model 1.

**Results:**

Algorithmic management was positively associated with technostress (B = 0.38, *p* < 0.001), whereas techno-related control was negatively associated with technostress (B = −0.17, *p* < 0.001). In addition, the interaction between algorithmic management and techno-related control was negative and statistically significant (B = −0.12, SE = 0.038, *p* = 0.002, 95% CI [−0.19, −0.05]). The interaction explained an additional 2.1% of the variance in technostress (ΔR² = 0.021), indicating that the positive association between algorithmic management and technostress became weaker as techno-related control increased.

**Discussion:**

These findings suggest that techno-related control may represent a meaningful, although modest, boundary condition in the relationship between algorithmic management and technostress and may help explain differences in how sport science professionals experience technology-mediated managerial demands.

## 1 Introduction

Advances in digital technologies have increasingly transformed performance evaluation, training planning, and managerial decision-making in sport sciences into data-driven processes. Artificial intelligence, wearable technologies, and performance analysis systems enable data not only to be collected but also to be actively integrated into decision-making processes in sport settings (1). This transformation has reshaped information management, work practices, and managerial processes within sport organizations (2). The impact of digital transformation in sport organizations extends beyond technological infrastructure; organizational capacity, leadership, digital strategies, and employees’ digital competencies are also key components of this process (3). More broadly, research across organizational settings has shown that digital transformation can reshape operational processes, decision-making structures, service delivery, and professional roles, highlighting that the organizational consequences of advanced digital technologies extend beyond any single sector (4). Evidence on the influence of digital technologies on information management and organizational work processes further supports the managerial dimension of this transformation (5, 6). Therefore, as digital systems increasingly assume managerial functions in sport settings, the concept of algorithmic management has become increasingly relevant in the context of sport sciences.

Algorithmic management refers to the use of algorithm-based systems to perform or support managerial functions such as monitoring, goal setting, scheduling, performance evaluation, and work coordination (7). As these systems become increasingly integrated into organizational processes, they can reshape how employees receive information, organize their tasks, and respond to performance expectations (8). Although algorithmic management may improve efficiency and coordination, its effects on employees are not uniformly positive. Evidence suggests that such systems can both enable and constrain autonomy, while intensive algorithmic monitoring and standardization may increase work demands and reduce employee discretion (9). These characteristics are particularly relevant to technostress because algorithmic systems may increase employees’ exposure to technology-mediated monitoring, system-generated decisions, and data-driven performance demands. Cram et al. (10) demonstrated that algorithmic control can generate both challenge- and threat-related techno-stressors, suggesting that technologically mediated control may become a source of strain as digital demands intensify. Given the growing use of digital systems in performance monitoring and managerial decision-making in sport settings, examining the relationship between algorithmic management and technostress among sport science professionals provides a relevant extension of the existing literature.

Technostress can be understood as a technology-related job demand that may impair employee well-being when digital demands exceed available coping resources (11). Within this context, techno-related control may represent an important situational resource by providing employees with greater autonomy and flexibility in determining which technologies to use and how to use them in their work (12). This technology-specific form of control is conceptually consistent with previous research showing that perceived control over technology can facilitate more effective coping with technology-related demands (13). Similarly, autonomy and managerial support have been associated with stronger perceived behavioral control over information technology (14). However, the protective role of control may not be consistent across digitally mediated work environments. Evidence from gig work suggests that job autonomy does not always buffer the negative effects of technostress, indicating that the value of control may depend on how it is experienced within a specific work context (15). This issue is particularly relevant under algorithmic management, where technologically mediated control can simultaneously structure work and generate technostress (10). Therefore, it is important to examine whether techno-related control alters the relationship between algorithmic management and technostress.

Although technostress has begun to receive increasing attention in sport-related contexts, the existing evidence remains limited and fragmented. Research has shown that technology-related stress can contribute to emotional exhaustion among coaches (16), while self-tracking technologies may generate multiple techno-stressors in performance-oriented sport settings (17). At the same time, the expanding role of digital platforms and artificial intelligence in sport highlights the growing influence of technology on sport-related governance and decision-making processes (18). However, limited attention has been devoted to examining how algorithmic management may contribute to technostress among sport science professionals and whether techno-related control alters this relationship. Accordingly, this study aims to examine the relationship between algorithmic management and technostress among sport science professionals and to test the moderating role of techno-related control. In doing so, the study seeks to extend research on algorithmic management and technostress to the increasingly digitalized context of sport sciences.

### 1.1 Theoretical framework

Algorithmic management refers to the use of algorithm-based systems to support or perform managerial functions such as monitoring, coordination, evaluation, and decision-making. These systems can reshape work by simultaneously increasing managerial control and, in some cases, enabling greater autonomy, thereby reflecting the paradoxical nature of algorithmic management (9). From a sociotechnical perspective, algorithmic management is not limited to technological infrastructure; it also reflects organizational choices, power relations, and changing patterns of interaction between employees and management (8). This perspective is increasingly relevant to sport sciences as artificial intelligence and data-driven systems become more deeply integrated into sport organizations and managerial processes. AI applications are increasingly used to support decision-making, organizational planning, performance evaluation, and other operational functions in sport settings (19). At the same time, growing reliance on algorithmic systems raises concerns regarding transparency, accountability, bias, and the extent of human involvement in decision-making (20). Earlier sport management research has also emphasized both the potential value of AI and the organizational challenges associated with its integration (21). Accordingly, algorithmic management provides a relevant framework for examining how digitally mediated managerial practices may shape the work experiences of sport science professionals.

Technostress refers to stress arising from the use of digital technologies and is commonly associated with dimensions such as techno-overload, techno-invasion, techno-complexity, techno-insecurity, and techno-uncertainty. Meta-analytic evidence indicates that these technology-related demands can negatively affect both psychological and behavioral outcomes, particularly when digital systems increase workload, complexity, and constant connectivity (22). In sport sciences, technostress has become increasingly relevant as digital monitoring, self-tracking, performance analysis, and technology-supported communication become more deeply embedded in professional practice. Research in performance-oriented sport has identified stressors such as information overload, system complexity, unreliability, loss of control, continuous monitoring, and pressure associated with performance data (17, 23). Similar concerns have been observed among coaches, where technostress has been linked to emotional exhaustion, with techno-overload and techno-uncertainty emerging as particularly salient dimensions (16). Recent evidence from sports education also suggests that technostress may hinder innovation-related outcomes, while technological self-efficacy may help mitigate its negative effects (24). These findings indicate that technostress represents an important occupational issue in increasingly digitalized sport science environments.

According to the Job Demand–Control model, greater control over work can reduce the strain associated with high job demands by providing employees with greater decision latitude and an enhanced capacity to manage stressful situations (25). This perspective is also consistent with the Job Demands–Resources framework, in which control can function as a job resource that helps employees cope with technology-related demands (11). In technology-intensive work environments, techno-related control refers to employees’ perceived autonomy and flexibility in deciding which technologies to use and how to use them in their work (12). This technology-specific form of control is consistent with previous technostress research showing that perceived control over information technology can facilitate more effective coping with technology-related pressures (13). However, the buffering role of control is not consistent across digital work environments. Evidence indicates that job control does not always reduce the adverse effects of techno-stressors (26), while some studies have reported non-significant interactions between job control and technology-related demands (27). Similarly, job autonomy has not consistently moderated the negative effects of technostress on well-being (15). These mixed findings suggest that the protective role of control may depend on the characteristics of the digital work environment. Therefore, examining techno-related control as a moderator may help clarify whether technology-specific autonomy and flexibility alter the relationship between algorithmic management and technostress among sport science professionals.

### 1.2 Hypothesis development

In sport-related work environments, algorithmic management is increasingly embedded in activities such as performance monitoring, scheduling, evaluation, and decision support. Although these systems may facilitate coordination and information processing, they may also intensify professionals’ exposure to digitally mediated work demands. Continuous data capture, automated feedback, system-generated performance expectations, and technology-mediated monitoring can increase the cognitive and psychological demands associated with technology use. Previous research has shown that algorithmic forms of control may contribute to both challenge- and threat-related techno-stressors (10). In sport science settings, where performance data and digital monitoring are increasingly integrated into professional practice, greater exposure to algorithmic management may therefore be associated with higher levels of technostress:

*H_1_*. Higher exposure to algorithmic management is positively associated with technostress among sport science professionals.

The association between algorithmic management and technostress may also depend on the degree of discretion professionals perceive in their use of workplace technologies. In the present study, techno-related control refers specifically to autonomy and flexibility in choosing which technologies to use and determining how they are used in work tasks. It therefore represents control over technology use rather than control exercised through algorithmic systems. This distinction is important because greater discretion over technology use may allow professionals to manage technology-related demands in ways that better fit their work practices. Previous research indicates that perceived control over technology can support coping with technostress (13), while Finstad et al. (12) conceptualize techno-related control as a technology-specific resource. However, evidence concerning the buffering role of job control and autonomy remains inconsistent across technology-intensive work settings (15, 26, 27). These mixed findings suggest that techno-related control may constitute an important boundary condition in the relationship between algorithmic management and technostress.

Conceptually, techno-related control was positioned as a moderator because it represents a technology-specific situational resource reflecting employees’ autonomy and flexibility in the use of workplace technologies (12). From the Job Demand–Control and Job Demands–Resources perspectives, such control may shape how employees respond to technology-related work demands rather than merely exerting an independent effect on strain (11, 25). Accordingly, employees exposed to similar levels of algorithmic management may experience different levels of technostress depending on the degree of discretion they retain over technology use. Although techno-related control may also be directly associated with technostress, the present study focuses on its potential role as a boundary condition that alters the strength of the association between algorithmic management and technostress. This conceptualization also distinguishes techno-related control from control exercised through algorithmic management: the former concerns employees’ perceived discretion in choosing and using workplace technologies, whereas the latter concerns the extent to which algorithmic systems structure, monitor, evaluate, or coordinate work (7, 12).

*H_2_*. Techno-related control moderates the relationship between algorithmic management and technostress among sport science professionals.

## 2 Method

The study employed a quantitative cross-sectional design to investigate the association between algorithmic management and technostress among professionals working in sport-related organizational settings. Particular attention was given to whether professionals’ perceived control over the technologies they use at work alters the strength of this association. The methodological approach therefore combined an assessment of the measurement properties of the study constructs with hypothesis testing based on a moderation model.

### 2.1 Participants and sport-organizational context

The study sample consisted of 372 professionals employed in sport-related organizations in which digital or artificial intelligence-supported systems were incorporated into managerial activities. The sample included coaches, sport managers, academics, performance specialists, strength and conditioning professionals, and exercise specialists. Participants represented a range of organizational settings, including sports clubs, universities, federations, performance centers, and fitness or sport centers. Participation was restricted to professionals who reported direct exposure to digitally mediated managerial practices in their workplace. Eligibility was established by determining whether digital or AI-supported systems were actively used within the participant’s organization for managerial functions such as monitoring work activities, evaluating performance, planning, scheduling, or supporting decision-making. Participants who did not report such organizational use were not included in the study sample. This criterion was intended to ensure that responses to the algorithmic management measures reflected relevant exposure to technology-supported managerial processes rather than general attitudes toward workplace technology or the routine use of digital tools for communication, data entry, or general administrative purposes. Exposure to algorithmic management was subsequently assessed using the Algorithmic Management Questionnaire, which captures employees’ perceived exposure to algorithmic monitoring, goal setting, scheduling, performance rating, and compensation practices. However, the study did not systematically record the commercial names, technical architecture, degree of automation, or specific algorithmic outputs generated by the systems used in each organizational setting. Accordingly, the findings should be interpreted as reflecting perceived exposure to algorithmic management practices as operationalized by the AMQ, rather than exposure to a single standardized algorithmic system or platform. The final sample comprised 229 men (61.6%) and 143 women (38.4%). Participants represented a broad age range, with the largest group being 28–35 years old (33.6%). Coaches constituted the largest occupational subgroup (30.9%), while sports clubs were the most frequently represented organizational setting (33.6%). Professional experience was also broadly distributed across the sample, with the largest proportion reporting 7–10 years of experience (22.8%).

The demographic and professional characteristics of the participants are presented in Table 1. Of the 372 participants, 61.6% were male and 38.4% were female. The largest age group was 28–35 years (33.6%), while 44.9% of the participants held a bachelor’s degree. Coaches constituted the largest professional group (30.9%), and sports clubs were the most frequently represented institution type (33.6%). In terms of professional experience, the largest proportion of participants had 7–10 years of experience (22.8%).

**Table 1:** Demographic and professional characteristics of participants.

Variable	Category	n	%
Gender	Female	143	38.4
	Male	229	61.6
Age	18–27 years	83	22.3
	28–35 years	125	33.6
	36–45 years	101	27.2
	46–54 years	47	12.6
	≥55 years	16	4.3
Education level	Bachelor’s degree	167	44.9
	Master’s degree	123	33.1
	Doctorate	82	22.0
Professional role	Coach	115	30.9
	Sport manager	66	17.7
	Academic	73	19.6
	Performance specialist	50	13.4
	Strength and conditioning professional	40	10.8
	Exercise specialist	28	7.5
Institution type	Sports club	125	33.6
	University	90	24.2
	Federation	54	14.5
	Performance center	47	12.6
	Fitness/sport center	56	15.1
Professional experience	0–3 years	64	17.2
	4–6 years	78	21.0
	7–10 years	85	22.8
	11–15 years	75	20.2
	≥16 years	70	18.8

### 2.2 Data collection procedure

To reduce the potential for common method bias during data collection, participation was voluntary and responses were collected anonymously. Participants were informed that there were no right or wrong answers and were encouraged to respond based on their own work experiences. These procedural measures were intended to reduce evaluation apprehension and encourage independent reporting. Nevertheless, because algorithmic management, techno-related control, and technostress were assessed using self-report measures obtained from the same respondents within the same data collection period, common method variance cannot be completely ruled out. Participants were recruited from sport-related organizational settings, including sports clubs, universities, federations, performance centers, and fitness or sport centers in Türkiye. Eligible professionals were invited to complete the study questionnaire after being informed about the purpose of the research and the eligibility criteria concerning exposure to digital or AI-supported systems used for managerial functions. Participation was voluntary, and questionnaire responses were not linked to personally identifying information. A total of 395 questionnaires were obtained. Of these, 23 were excluded because they were substantially incomplete and did not provide sufficient data for the planned analyses. Consequently, 372 complete questionnaires were retained for the final analyses. Because the total number of individuals who received the study invitation was not systematically recorded, a formal response rate could not be calculated.

### 2.3 Data collection instruments

*Technostress Creators Scale*: Technostress was assessed using the Turkish version of the Technostress Creators Scale (TSCS), whose validity and reliability were examined by Bulut et al. (28). The Turkish version consists of 22 items and comprises five dimensions of technostress. In the original Turkish validation study, confirmatory factor analysis supported the proposed factor structure, with acceptable model fit indices (χ^2^/df = 1.82, CFI = 0.902, IFI = 0.904, RMSEA = 0.071, SRMR = 0.030). Standardized factor loadings ranged from 0.591 to 0.912, while Cronbach’s alpha coefficients ranged from 0.79 to 0.83 across the subdimensions and the overall scale. Because the Turkish version was originally validated among health professionals, its psychometric performance was re-examined in the present sample of sport science professionals prior to hypothesis testing. The TSCS demonstrated satisfactory reliability and convergent validity in the present sample, with Cronbach’s α = 0.89, composite reliability (CR) = 0.90, and average variance extracted (AVE) = 0.56, supporting its use in the subsequent analyses.

*Algorithmic Management Scale:* Algorithmic management was assessed using the 20-item Algorithmic Management Questionnaire (AMQ) developed and validated by Parent-Rocheleau et al. (29). The AMQ assesses employees’ perceived exposure to algorithmic management across five dimensions: algorithmic monitoring, goal setting, scheduling, performance rating, and compensation. These dimensions represent a higher-order construct reflecting overall exposure to algorithmic management. Items are rated on a seven-point Likert scale ranging from 1 (totally disagree) to 7 (totally agree), consistent with the original instrument.

For use with Turkish-speaking participants, the AMQ was translated and cross-culturally adapted prior to data collection, as described in the subsequent section. The translated AMQ demonstrated satisfactory psychometric properties in the present Turkish sport science sample, with Cronbach’s α = 0.91, composite reliability (CR) = 0.92, and average variance extracted (AVE) = 0.58.

*Techno-related Control:* Techno-related control was assessed using the three-item Techno-related Control subscale of the Technology-Related Moderator Scale developed and preliminarily validated by Finstad et al. (12). This subscale assesses employees’ perceived autonomy and flexibility in technology use, including the extent to which they can choose which technologies to use, determine how to use them, and select the methods employed when using technology at work. Items were rated on a five-point Likert scale ranging from 1 (strongly disagree) to 5 (strongly agree). For use with Turkish-speaking participants, the subscale was translated and cross-culturally adapted prior to data collection, as described in the subsequent section. The translated subscale demonstrated satisfactory psychometric properties in the present Turkish sport science sample, with Cronbach’s α = 0.87, composite reliability (CR) = 0.88, and average variance extracted (AVE) = 0.59.

#### 2.3.1 Translation and cross-cultural adaptation of the AMQ and techno-related control subscale

As previously validated Turkish versions of the Algorithmic Management Questionnaire (AMQ) and the Techno-related Control subscale were not available, the instruments were translated and linguistically adapted from English into Turkish prior to data collection. The procedure was informed by established recommendations for the translation and cross-cultural adaptation of self-report measures (30–32).

First, the original English items were independently translated into Turkish by bilingual individuals proficient in both English and Turkish. The resulting translations were compared item by item, with particular attention to semantic, conceptual, and contextual equivalence rather than literal word-for-word correspondence. Discrepancies were discussed and resolved through consensus to produce a reconciled Turkish version.

The reconciled Turkish items were subsequently translated back into English by bilingual individuals who had not been involved in the initial forward translation. The back-translated items were compared with the original English versions to identify potential changes in meaning or construct representation. Any discrepancies were reviewed and resolved through discussion, and the Turkish wording was revised where necessary.

Finally, the translated items were reviewed for linguistic clarity, comprehensibility, conceptual equivalence, and applicability to Turkish sport-related professional and organizational settings. Only minor linguistic or contextual modifications were permitted, and no changes were made to the underlying constructs, number of items, dimensional structure, or response formats of the original instruments. The finalized Turkish versions were subsequently used for data collection.

To further establish their suitability for the present sample, the factorial structure, internal consistency, composite reliability, and convergent validity of both translated measures were examined before hypothesis testing. The Turkish AMQ demonstrated satisfactory psychometric properties (Cronbach’s α = 0.91, CR = 0.92, AVE = 0.58), as did the Techno-related Control subscale (Cronbach’s α = 0.87, CR = 0.88, AVE = 0.59).

### 2.4 Measurement model

Before testing the structural relationships among the study variables, the psychometric performance of the measures was examined in the sport science sample. Confirmatory factor analysis was conducted to determine whether the theoretically specified factor structures of algorithmic management, technostress, and techno-related control were adequately represented in the present data. Model fit was judged using a combination of absolute and incremental fit indicators, including CFI, TLI, IFI, RMSEA, and sRMR. Reliability was evaluated using Cronbach’s alpha and composite reliability (CR), while convergent validity was assessed through average variance extracted (AVE). The measurement results were used to establish whether the constructs demonstrated sufficient reliability and validity for subsequent moderation analysis (Table 2).

**Table 2:** Confirmatory factor analysis fit indices of the measurement model for the proposed moderation framework (*N* = 372).

Fit index	Estimated value	Reference threshold	Evaluation
χ^2^	1826.72	-	-
df	932	-	-
χ^2^/df	1.96	≤ 3.00	Acceptable fit
CFI	0.941	≥ 0.90	Good fit
TLI	0.933	≥ 0.90	Good fit
IFI	0.942	≥ 0.90	Good fit
RMSEA	0.051	≤ 0.08	Acceptable fit
sRMR	0.044	≤ 0.08	Acceptable fit

The confirmatory factor analysis results indicated that the measurement model demonstrated an acceptable fit in the sport science sample, χ^2^(932) = 1826.72, *p* < 0.001, χ^2^/df = 1.96. The incremental fit indices were above the recommended threshold (CFI = 0.941, TLI = 0.933, IFI = 0.942). In addition, RMSEA (0.051) and sRMR (0.044) were within acceptable limits. These findings supported the adequacy of the proposed measurement model for the study variables in the present sample.

### 2.5 Analytical strategy

Analyses were performed using IBM SPSS Statistics 26.0, AMOS 26.0, and Hayes’ PROCESS Macro 4.2. Confirmatory factor analysis was conducted to evaluate the measurement properties of the study constructs, followed by reliability and validity assessments. The hypothesized moderation model was tested using PROCESS Model 1, with algorithmic management specified as the predictor (X), techno-related control as the moderator (W), and technostress as the outcome variable (Y). Continuous predictors were mean-centered before computation of the interaction term (X × W). Skewness and kurtosis were examined to assess distributional characteristics, and variance inflation factors were inspected for potential multicollinearity. Because the study hypotheses concerned overall algorithmic management and overall technostress rather than their individual dimensions, composite scores were used in the moderation analysis after the adequacy of their measurement structures, reliability, and validity had been established. Nevertheless, both constructs are multidimensional; therefore, dimension-specific relationships were examined separately as supplementary analyses.

### 2.6 Ethical considerations

Ethical approval for the study was obtained from the Hitit University Non-Interventional Research Ethics Committee. The study was reviewed and approved at the committee meeting held on 25 March 2026, with Decision No. 2026–09 and Application No. 2026–0128. The ethics committee confirmed that the research protocol was ethically appropriate.

## 3 Results

The results indicated that algorithmic management was positively associated with technostress, whereas techno-related control was negatively associated with technostress. Algorithmic management also showed a modest positive association with techno-related control. These findings were consistent with the proposed model, in which algorithmic management was hypothesized to be positively associated with technostress, while techno-related control may function as a technology-specific resource (Table 3).

**Table 3:** Descriptive statistics and Pearson correlations for the sport science sample.

Variables	Mean	SD	1	2	3
1. Algorithmic management	3.36	0.81	1.000		
2. Techno-related control	3.44	0.76	0.26**	1.000	
3. Technostress	3.21	0.74	0.41**	−0.19**	1.000

**p < 0.01, two-tailed.

As a supplementary analysis, dimension-level correlations between algorithmic management and technostress were examined to assess whether the aggregate association was broadly reflected across their constituent dimensions. As shown in Table 4, all associations were positive and statistically significant (all *p* < 0.001), with coefficients ranging from r = 0.341 to r = 0.405. Although the magnitude of the relationships varied across specific dimensions, the overall pattern was consistent with the positive construct-level association between algorithmic management and technostress. These findings indicate that the construct-level analysis captures a broadly consistent positive pattern while also showing that the strength of the associations varies across specific subdimensions (Table 5).

**Table 4:** Dimension-level correlations between algorithmic management and technostress.

Algorithmic management dimension	Techno-overload	Techno-invasion	Techno-complexity	Techno-insecurity	Techno-uncertainty
Algorithmic Monitoring	0.397***	0.385***	0.396***	0.370***	0.398***
Goal Setting	0.384***	0.370***	0.374***	0.366***	0.390***
Scheduling	0.392***	0.384***	0.388***	0.363***	0.391***
Performance rating	0.401***	0.386***	0.395***	0.372***	0.405***
Compensation	0.362***	0.348***	0.370***	0.341***	0.367***

***p < 0.001, two-tailed.

**Table 5:** Reliability and validity indicators for the sport science sample.

Construct	Standardized factor loadings	Cronbach’s α	CR	AVE	Evaluation
Algorithmic Management	0.68–0.84	0.91	0.92	0.58	Satisfactory
Technostress	0.65–0.83	0.89	0.90	0.56	Satisfactory
Techno-related control	0.72–0.82	0.87	0.88	0.59	Satisfactory

CR, composite reliability; AVE, average variance extracted.

The measurement results indicated satisfactory reliability and convergent validity for the study variables in the sport science sample. Standardized factor loadings were acceptable across the measurement model, ranging from 0.68 to 0.84 for algorithmic management, 0.65 to 0.83 for technostress, and 0.72 to 0.82 for techno-related control. Cronbach’s alpha, composite reliability, and average variance extracted values were also within acceptable ranges for all constructs. Together with the confirmatory factor analysis results presented in Table 2, these findings supported the adequacy of the measurement model.

Discriminant validity was assessed using the Fornell–Larcker criterion. As shown in Table 6, the square root of the AVE for each construct was greater than the absolute value of its correlations with the other constructs. Specifically, the square roots of the AVE were 0.762 for algorithmic management, 0.768 for techno-related control, and 0.748 for technostress. These findings supported adequate discriminant validity among the three constructs.

**Table 6:** Discriminant validity based on the Fornell–Larcker criterion.

Construct	1	2	3
1. Algorithmic management	**0.762**		
2. Techno-related control	0.260	**0.768**	
3. Technostress	0.410	−0.190	**0.748**

Diagonal values in bold represent the square root of the average variance extracted (AVE), whereas off-diagonal values represent correlations between the constructs.

### 3.1 Moderation analysis

Technostress was specified as the dependent variable (Y), algorithmic management as the predictor (X), and techno-related control as the moderator (W). The interaction term between algorithmic management and techno-related control (X × W) was included in the PROCESS Model 1 analysis (Figure 1).

**Figure 1:**
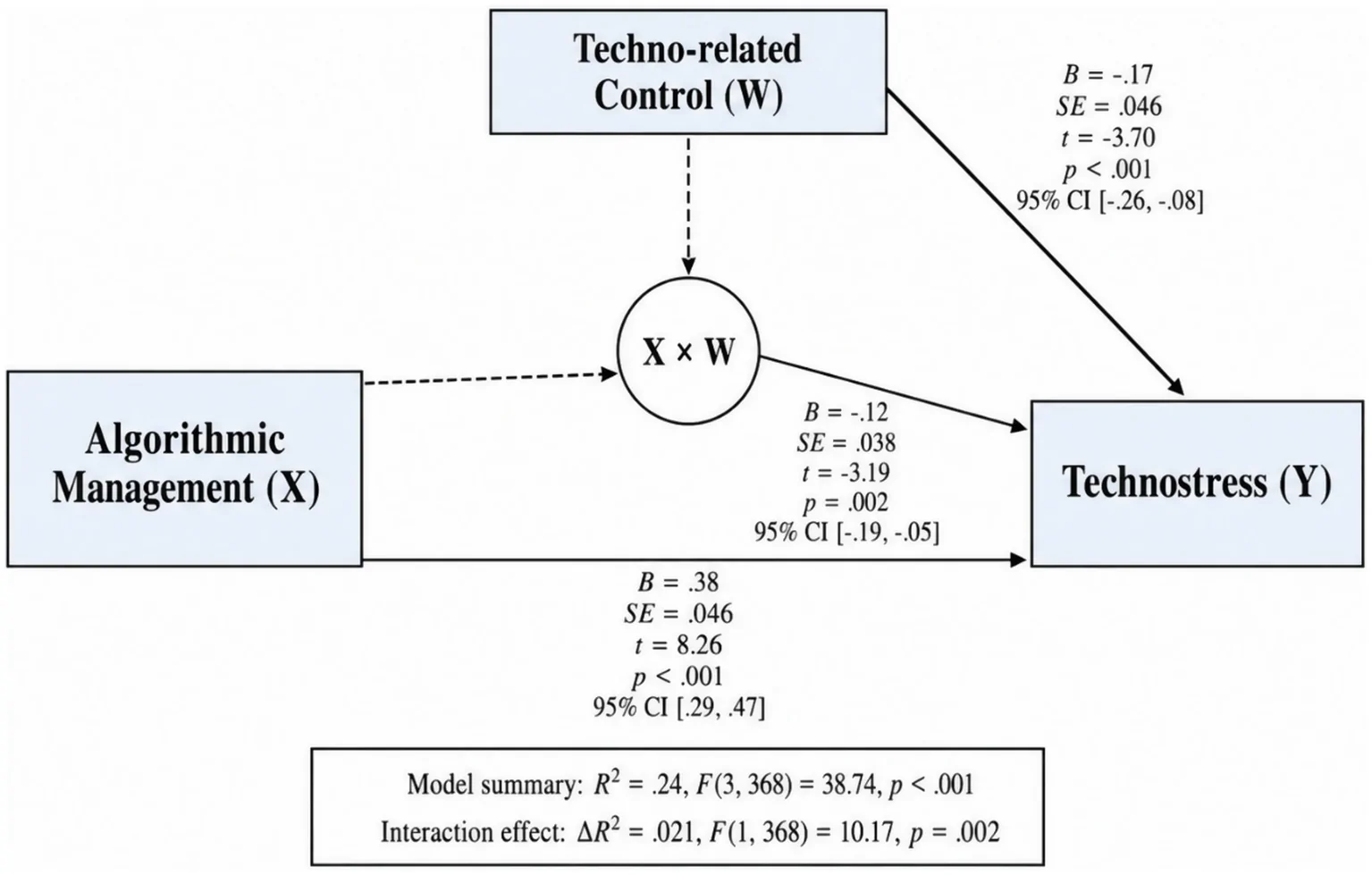
Path diagram of the moderating role of techno-related control in the relationship between algorithmic management and technostress among sport science professionals.

The moderation analysis showed that algorithmic management was positively associated with technostress (B = 0.38, *p* < 0.001), whereas techno-related control was negatively associated with technostress (B = −0.17, *p* < 0.001). The interaction between algorithmic management and techno-related control was negative and statistically significant (B = −0.12, SE = 0.038, t = −3.19, *p* = 0.002, 95% CI [−0.19, −0.05]). The interaction explained an additional 2.1% of the variance in technostress (ΔR^2^ = 0.021), indicating that the positive association between algorithmic management and technostress became weaker as techno-related control increased (Table 7).

**Table 7:** PROCESS model 1 moderation analysis results.

Predictor	B	SE	t	*p*	95% CI Lower	95% CI Upper
Constant	3.21	0.038	84.47	< 0.001	3.14	3.28
Algorithmic management (X)	0.38	0.046	8.26	< 0.001	0.29	0.47
Techno-related control (W)	−0.17	0.046	−3.70	< 0.001	−0.26	−0.08
X × W	−0.12	0.038	−3.19	0.002	−0.19	−0.05

Model summary: R^2^ = 0.24, F(3, 368) = 38.74, *p* < 0.001; interaction effect: ΔR^2^ = 0.021, F(1, 368) = 10.17, *p* = 0.002.

A *post hoc* power analysis was conducted for the moderation effect. Based on the final sample size (N = 372), an alpha level of 0.05, and an effect size calculated from the observed incremental variance explained by the interaction term (ΔR^2^ = 0.021; f^2^ ≈ 0.028), the achieved statistical power was approximately 0.89. This result indicates that the current sample provided adequate statistical power to detect the observed moderation effect (Table 8).

**Table 8:** Conditional effects of algorithmic management on technostress at different levels of techno-related control.

Techno-related control	Value	Conditional effect (B)
Low (−1 SD)	2.68	0.471
Mean	3.44	0.380
High (+1 SD)	4.20	0.289

Conditional effects were calculated at one standard deviation below the mean, the mean, and one standard deviation above the mean of techno-related control.

To further interpret the significant interaction, conditional effects of algorithmic management on technostress were examined at low (−1 SD), mean, and high (+1 SD) levels of techno-related control. The positive association between algorithmic management and technostress was strongest at low levels of techno-related control (B = 0.471), decreased at the mean level (B = 0.380), and was weakest at high levels of techno-related control (B = 0.289). Thus, the positive association between algorithmic management and technostress became progressively weaker as techno-related control increased (Figure 2).

**Figure 2:**
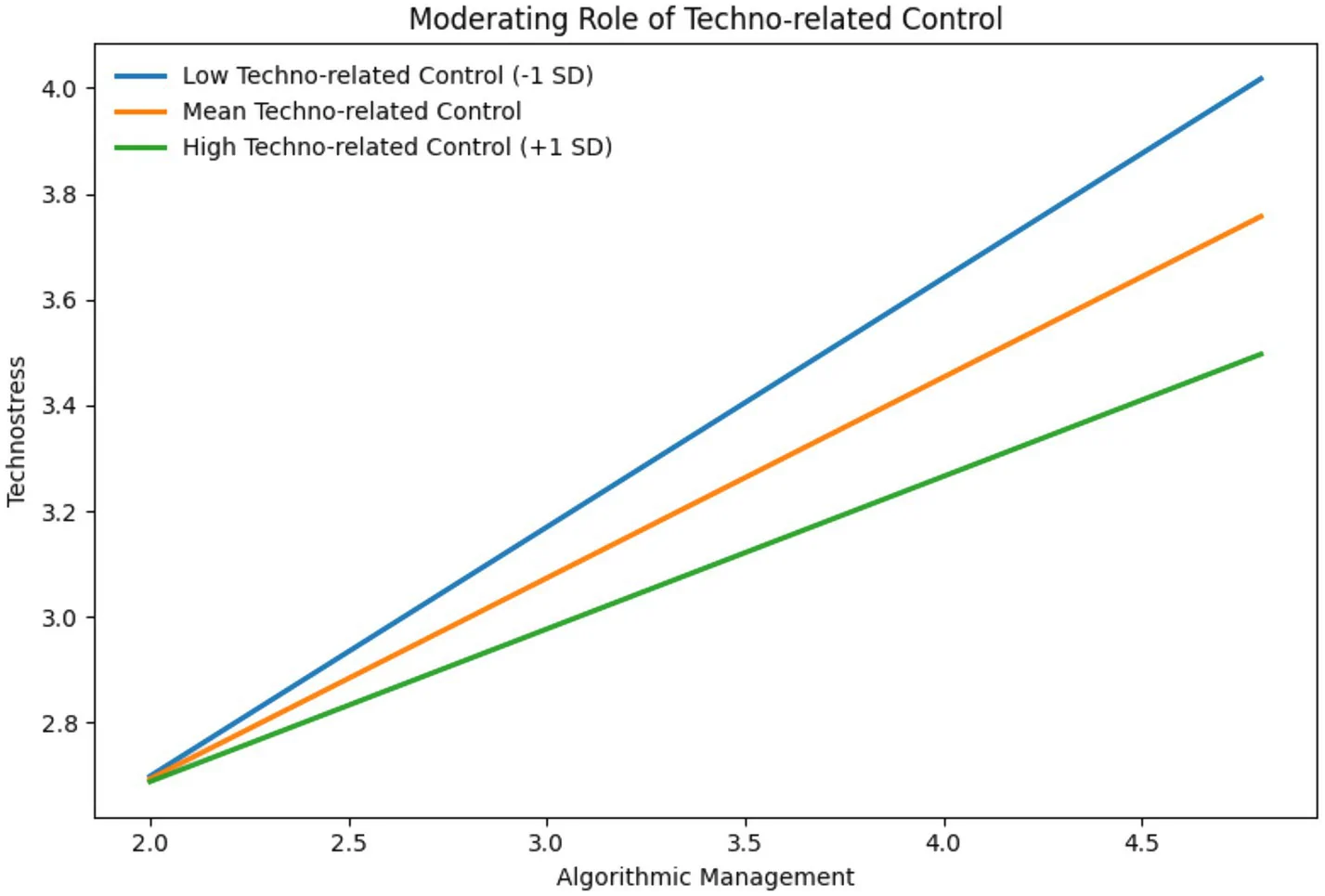
Moderating role of techno-related control in the relationship between algorithmic management and technostress.

The figure illustrates that the predicted positive relationship between algorithmic management and technostress becomes weaker as techno-related control increases. The association is strongest at low levels of techno-related control and comparatively weaker at high levels. The plotted lines were derived from the coefficients reported in the moderation model (Table 9).

**Table 9:** Group comparisons of the main study variables across professional role and institution type.

Grouping variable	Outcome variable	df	F	*p*	η^2^
Professional role	Algorithmic management	5, 366	0.47	0.799	0.006
Professional role	Techno-related control	5, 366	1.38	0.231	0.019
Professional role	Technostress	5, 366	0.42	0.835	0.006
Institution type	Algorithmic management	4, 367	0.37	0.833	0.004
Institution type	Techno-related control	4, 367	0.87	0.483	0.009
Institution type	Technostress	4, 367	1.62	0.169	0.017

No statistically significant differences were observed across professional-role or institution-type groups for algorithmic management, techno-related control, or technostress (all *p* > 0.05). Effect sizes were small, indicating limited between-group variability in the main study variables (Table 10).

**Table 10:** Robustness analysis of the moderating effect after controlling for professional role and institution type.

Predictor	B	SE	t	*p*	95% CI Lower	95% CI Upper
Algorithmic management	0.426	0.043	9.95	< 0.001	0.342	0.511
Techno-related control	−0.175	0.046	−3.81	< 0.001	−0.266	−0.085
Algorithmic management × techno-related control	−0.172	0.058	−2.99	0.003	−0.285	−0.059

Professional role and institution type were included as categorical covariates. Continuous predictors were mean-centered prior to calculation of the interaction term.

After controlling for professional role and institution type, the interaction between algorithmic management and techno-related control remained negative and statistically significant (B = −0.172, SE = 0.058, t = −2.99, *p* = 0.003, 95% CI [−0.285, −0.059]), indicating that the moderation effect remained significant after adjustment for occupational and institutional differences.

### 3.2 Diagnostic checks

Additional diagnostic checks were conducted to evaluate potential sources of bias in the estimated relationships. Harman’s single-factor test indicated that the first unrotated factor accounted for 36.4% of the total variance, suggesting that the variance was not dominated by a single common factor. As an additional diagnostic assessment of potential common method bias, full-collinearity variance inflation factors were examined for the three principal constructs. The resulting VIF values were 1.357 for algorithmic management, 1.112 for techno-related control, and 1.286 for technostress. All values were substantially below the suggested threshold of 3.3 for identifying potentially problematic full collinearity (33). Considered together with the Harman single-factor test, these results did not indicate that common method variance was likely to dominate the observed relationships. However, these diagnostics cannot establish the absence of common method bias. Because all principal variables were assessed using self-report measures obtained from the same respondents within the same data collection period, the possibility of common method variance cannot be completely excluded. Separately, conventional multicollinearity diagnostics for the predictors included in the moderation model indicated no problematic overlap, with VIF values remaining below 2.5. No substantial departures from linearity were identified in the preliminary assessment of the moderation model.

## 4 Discussion

The positive association observed between algorithmic management and technostress suggests that algorithmically mediated work processes may represent an important source of technology-related strain for sport science professionals. This interpretation is consistent with Cram et al. (10), who showed that algorithmic control can generate both challenge- and threat-related techno-stressors. From a work-design perspective, such systems may increase exposure to monitoring, workload, and performance demands while simultaneously narrowing autonomy and decision latitude (7). Similar concerns have been reported across other work settings, where algorithmic management has been linked to psychosocial risks and mental strain (34, 35). Within sport environments, these processes may be particularly salient because digital monitoring and data-based performance evaluation are increasingly integrated into routine professional practice. Werner and Bischof (17) identified information overload, system complexity, continuous monitoring, and loss of control as important technostress factors in performance-oriented sport, while Michalik and Schermuly (16) reported an association between technostress and emotional exhaustion among coaches. At the same time, algorithmic control should not be interpreted as uniformly detrimental. Zhu et al. (36) showed that perceived algorithmic control may produce both constructive and adverse responses, whereas Zhao et al. (37) demonstrated that algorithmic control can function as both a challenge and a hindrance stressor. Taken together, the present findings indicate that greater exposure to algorithmic management is associated with higher technostress among sport science professionals, while also suggesting that the way algorithmic systems are implemented and experienced may shape the intensity of this relationship.

The negative association between techno-related control and technostress suggests that greater discretion over workplace technology may be linked to lower levels of technology-related strain among sport science professionals. This interpretation is consistent with Pirkkalainen et al. (13), who showed that perceived control over technology can support more effective coping with technostress, and with Salanova et al. (38), who emphasized the role of control and decision latitude in reducing technology-related strain. More recent findings have also pointed to the potential protective value of perceived control. Meier et al. (39) identified perceived control as a resilience factor associated with lower stress responses, while Fielder et al. (40) showed that a stronger sense of control can buffer subsequent stress. At the same time, the literature does not indicate a uniformly protective effect of control across technology-intensive work settings. Kaltenegger et al. (27) reported no clear buffering interaction between job control and techno-stressors, while Rohwer et al. (26) suggested that control-related resources may operate differently depending on the type of technological demand. Similarly, Datta et al. (41) showed that autonomy can have context-dependent effects in technology-intensive work settings. Weinert (42) further conceptualized perceived control as a secondary appraisal mechanism that may influence whether techno-stressors are experienced as challenges or hindrances. In this context, the present findings indicate that techno-related control is associated with lower technostress among sport science professionals, while also reinforcing the view that the relevance of control depends on how technology-related demands are structured and experienced within the work environment.

The significant interaction between algorithmic management and techno-related control indicates that the strength of the association between algorithmic management and technostress varies according to the level of control professionals perceive over workplace technology. More specifically, the positive relationship between algorithmic management and technostress became weaker as techno-related control increased, providing support for H_2_. This pattern is consistent with research emphasizing the role of autonomy-related resources in shaping responses to algorithmically mediated work. Peng et al. (43) showed that algorithmic management may influence burnout through autonomy-related mechanisms, while Deng et al. (44) demonstrated that algorithmic autonomy support can shape workers’ stress responses. In a similar vein, Wan et al. (45) highlighted the context-dependent role of job autonomy in gig workers’ well-being, and Felix et al. (46) showed that the effects of algorithmic management may vary according to the fit between workers’ autonomy preferences and the characteristics of algorithmically managed work. Taken together, these findings support the interpretation that techno-related control may operate as a context-sensitive resource associated with differences in the strength of the relationship between algorithmic management and technostress.

More recent studies further support the view that control-related resources may shape how algorithmically mediated work is experienced. Zha et al. (47) identified job autonomy as an important mechanism linking algorithmic management to worker well-being, while Wang et al. (48) demonstrated that associations involving algorithmic HRM systems and self-efficacy vary according to the configuration of algorithmic monitoring, control, and techno-stressors. Huo et al. (49) likewise showed that the relationship between algorithmic control and well-being is not uniform, but differs according to contextual and individual conditions. Finstad et al. (12) further emphasized that technology-specific control resources may be more informative than broad job-control constructs in explaining resilience to technostress. In the present study, higher techno-related control was associated with a weaker positive relationship between algorithmic management and technostress. However, the interaction explained only a modest proportion of additional variance (ΔR^2^ = 0.021). Accordingly, techno-related control should be interpreted as a meaningful but limited boundary condition rather than as a dominant explanatory factor in this relationship.

## 5 Theoretical contributions

The theoretical contribution of this study lies in distinguishing technology-related discretion from algorithmically imposed control within digitally mediated work environments. Rather than treating control as a single, uniform construct, the findings suggest that employees’ discretion over technology use may represent a distinct technology-specific resource. This distinction extends the Job Demand–Control and Job Demands–Resources perspectives by indicating that technology-specific control may be particularly relevant when examining technology-mediated work demands. The significant moderation effect further suggests that techno-related control may serve as a meaningful, although modest, boundary condition in the association between algorithmic management and technostress among sport science professionals.

## 6 Practical implications

The findings offer several practical considerations for sport organizations and institutions using digital and algorithm-supported systems. Organizations may benefit from considering not only the implementation of advanced technologies but also the extent to which sport science professionals perceive autonomy and flexibility in technology-related work processes. In this context, human-centered implementation practices, opportunities for user participation, adequate technical support, and clear communication regarding digital systems may be relevant considerations when designing technology-mediated work environments. However, because the present study was cross-sectional, these findings do not establish that increasing professional discretion or implementing such practices will necessarily reduce technostress. Their potential effects should therefore be examined in longitudinal and intervention-based research.

## 7 Limitations and future research

Several limitations should be considered when interpreting the findings. First, the sample included professionals from diverse occupational and organizational settings; therefore, the results reflect general patterns rather than profession-specific effects. Future studies should examine more homogeneous professional groups and different levels of technological integration. Second, algorithmic management and technostress were examined primarily at the overall construct level. Although supplementary analyses supported a broadly consistent pattern across dimensions, future research should investigate dimension-specific relationships. Third, all main variables were assessed using self-report measures collected from the same respondents, meaning that common method variance and response bias cannot be completely ruled out. Future studies should incorporate objective or multi-source indicators of technology use and algorithmic management. Finally, the cross-sectional design prevents causal and temporal conclusions. Longitudinal and repeated-measures studies are needed to examine how algorithmic management, techno-related control, and technostress are related over time and to test the generalizability of these relationships across different sport organizations and national contexts.

## 8 Conclusion

This study examined the relationship between algorithmic management and technostress among sport science professionals and investigated the moderating role of techno-related control. The findings showed that algorithmic management was positively associated with technostress, whereas techno-related control was negatively associated with technostress and significantly weakened the positive association between algorithmic management and technostress. These findings supported both proposed hypotheses. Supplementary and robustness analyses supported the consistency of the main findings across dimensions and after adjustment for professional role and institution type. Overall, the results suggest that techno-related control may represent an important boundary condition in the relationship between algorithmic management and technostress among sport science professionals. Future research should examine these relationships longitudinally and across different professional, organizational, and national contexts.

## Data Availability

The raw data supporting the conclusions of this article will be made available by the authors, without undue reservation.
